# Isokinetic Strength Ratios: Conventional Methods, Current Limits and Perspectives

**DOI:** 10.3389/fphys.2019.00567

**Published:** 2019-05-21

**Authors:** Maryne Cozette, Pierre-Marie Leprêtre, Clare Doyle, Thierry Weissland

**Affiliations:** ^1^Laboratoire de Recherche Adaptations Physiologiques à l'Exercice et Réadaptation à l'Effort, UFR-STAPS, Université de Picardie Jules Verne, Amiens, France; ^2^Institut de Formation en Masso-Kinésithérapie (IFMK), Hôpital La Musse, Fondation Hospitalière La Renaissance Sanitaire, Université de Rouen, Saint Sébastien de Morsent, France

**Keywords:** strength, peak torque, muscle balance, rehabilitation, injury

Muscular performance and the risk of injury may depend on the balance of opposing muscle groups (Tam et al., 2017). It is a common and useful practice in clinical set up to assess the relative balance of opposing muscle groups around a joint by comparing strength ratios of agonist and antagonist muscle groups (Sapeda, 1990). This ratio is classically explored using an isokinetic dynamometer (Dvir, 2004). Isokinetic technology has become a popular method in order to assess muscle function in both clinical and research settings (Drouin et al., 2004). The concept is based on the measure of muscle torque recorded during isolated joint exercise within which angular velocity remains constant. Isokinetic dynamometers provide an accommodating resistance equal and opposite to the muscular forces applied for maximum external-force output with changing positions of the joint (Sapeda, 1990). The agonist-antagonist muscle balance is explored through different isokinetic ratios. First, the conventional ratio represents a concentric antagonist/agonist ratio (Evangelidis et al., 2015). The second ratio used is called the functional ratio (eccentric antagonist/concentric agonist) and it has been developed especially for the evaluation of athletes (Mont et al., 1994; Scoville et al., 1997). Other authors have proposed a mixed functional ratio, combining two different angular velocities (Croisier et al., 2002; Forthomme et al., 2013; Evangelidis et al., 2015). However, in clinical practice, the isokinetic ratios are calculated systematically with respective simple peak torque (PT) measurements for agonist and antagonist muscle groups. Some sectorial methods have emerged and appear to be conceptually very promising, but are little exploited and remain exclusive to the field of research. The development of these methods would be of interest to appreciate the muscle balance in the field of rehabilitation and sport. The objective of this opinion article letter is to provide critical point of view about the classical method associated with PT, and to present the emerging methods.

Classically, the isokinetic antagonist/agonist ratios are calculated with simple PT measurements for agonist and antagonist muscle groups (Figure 1). PT-ratios have a limited ability to characterize the entire torque variations inherent to the length-tension relationship because the PT corresponds to the torque generated at a single point of the entire ROM (Dvir, 2004; Amaral et al., 2014; Eustace et al., 2017). Furthermore, the respective agonist and antagonist PT values are not reached at the same angulation (Croisier and Crielaard, 1999). The PT value depends on biomechanical properties such as torque-angle relationship (Stefanska, 2006). The PT is reached at the optimal angle (α) for a specific joint position (Amaral et al., 2014). This angle is related to physiological factors such as optimal muscle length (length-tension relationship) and mechanical factors (changes in the angle of insertion or lever arm during rotatory motion) during isokinetic assessment (Amaral et al., 2014; Cibulka et al., 2014). For example, α has been reported at ~70° for knee extensors in the concentric modality and at ~35° for knee flexors in the eccentric modality (Coombs and Garbutt, 2002; Small et al., 2010). Furthermore, α is a low reliable (Atkinson and Nevill, 1998) isokinetic parameter to assess shoulder rotators (ICC = 0.51–0.93) (Papotto et al., 2016) and knee flexors and extensors (ICC = 0.46–0.88) (Bernard et al., 2012). Moreover, α can be affected by several factors, including test conditions For example, α was less reliable at high isokinetic velocity for knee extensor muscles (Bernard et al., 2012). Croisier and Crielaard (1999) highlighted the limitations of the unique use of PT by isokinetic qualitative analysis. Torque-angle relationship trend curves can help to detect anomalies such as reflex inhibition associated with joint pain (e.g., non-operated ligament lesion), major muscle atrophy associated with insufficient neuromuscular recruitment (e.g., three months post-operative after anterior cruciate ligament reconstruction), or a biomechanical disturbance (e.g., patella femoral syndrome) (Croisier and Crielaard, 1999). These anomalies detected in the analysis of curves are not necessarily identified in the analysis of traditional quantitative data, including the study of the PT. In addition, the PT-ratios are known to be extremely variable and low reliable (Atkinson and Nevill, 1998; Hopkins, 2000), especially for PT-ratios of shoulder rotators (ICC = 0.48–0.83; CoV = 7.5–12.9%) (Edouard et al., 2011). This may be partially attributable to the fact that ratios are composed of two measurements, each of which may vary in two directions with repeated testing or not change at all. The low reliability could be attributable to accumulated errors in the measurement process, especially when used without correction for gravity (Edouard et al., 2011).

**Figure 1:**
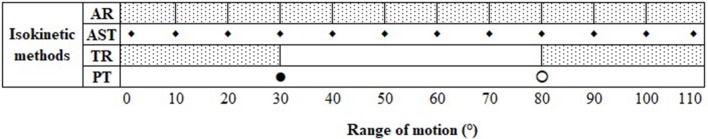
Illustration of four isokinetic methods used to calculate agonist-antagonist ratios. Peak Torque (PT) method is the ratio between PT of agonist (•) and PT of antagonist (○) independently of the angular position; Terminal Ranges (TR) method is the ratio between mean torque 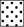 of agonist and antagonist at the 15° terminal ranges; Angle Specific Torque (AST) is the ratio between torque (♦) of agonist and antagonist at every 10° of the ROM; and Angular Ranges (AR) method is the ratio between mean torque 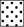 of agonist and antagonist by angular range of 10°. The angles chosen in this illustration are examples.

Together, these observations raise the question about the interest of the PT-ratios in the exploration of muscle balance. The lack of information taken into account across the entire ROM, the optimal angle differences between agonist and antagonist muscle groups, and the lack of reliability of these PT-ratios have led to the development of complementary quantitative methods. Thus, three methods have been developed to calculate isokinetic ratios based not on two peak torque values, but on angular sectors.

The first sector-dependent calculation method is the terminal ranges (TR) method. Some authors have studied the TR of the ROM for the testing of shoulder rotator muscles (Figure 1) (Scoville et al., 1997; Yildiz et al., 2006). The concept was developed from the idea that the overhead athlete must have an adequate functional ratio for dynamic stability and an optimal function, especially in the range where the antagonist is functioning as a brake: the terminal range. Yildiz et al. (2006) showed significant differences between the dominant and non-dominant shoulder for the functional ratios calculated from TR method. However, this method constitutes a methodological bias because the measured data concerns non-isokinetic ranges. The isokinetic device requires an acceleration phase to the attainment of pre-selected velocity, followed by a deceleration phase. As a result, such periods of acceleration and deceleration may increase the number of errors in the analysis due to the integration of non-isokinetic data. Indeed, machine-offered resistance requires the attainment and the upkeep of velocity pre-selected. Therefore, limb acceleration offers no overload other than the weight of the limb and the dynamometer attachment. Deceleration offers machine resistance but is no longer isokinetic and therefore contributes only artifacts to data analysis. According to Brown et al. (1995), this inability to attain of the pre-selected velocity negates the effect of machine-offered resistance. The acceleration and deceleration phases increase significantly with velocity increase, and therefore reduce the isokinetic range (Brown et al., 1995).

The second sectorial method is called angle specific torque (AST). The AST method has been developed to better reflect the antagonistic function as part of the exploration of the flexor and extensor muscles of the knee (Evangelidis et al., 2015). It consists of measuring the muscle torque at specific joint angles (Figure 1). The studies showed differences between agonist and antagonist muscle torque, and the AST-ratios were not constant during the ROM (Coombs and Garbutt, 2002; Cohen et al., 2015; El-Ashker et al., 2017). Cohen et al. (2015) highlighted significant declines in hamstring torque at 10°, 50°, 60°, 90° and a decrease of 22% of the functional ratio (eccentric hamstring/concentric quadriceps) at 10° of knee flexion after simulated soccer activity, which could increase vulnerability to hamstring injury. The AST method has also been used in the exploration of shoulder rotator muscle function. Ruas et al. (2014) showed that AST-ratios were not constant during the ROM. No differences were found between the AST and PT methods for conventional ratios. Their results show that it is not necessary to use the AST method to calculate this ratio. Conversely, functional AST-ratio was different to functional PT-ratio at several angles at 60°s^−1^ and 180°s^−1^. These different studies using the AST-method demonstrated that PT-ratios may result in misinterpretation of the muscle balance if a more specific approach is not utilized.

Finally, the angular range (AR) method was initiated in the exploration of the knee flexor/extensor muscles (Kellis and Katis, 2007). The AR method consists of recording the torque on all the ROM and averaging it by an angular range (Figure 1). Kellis and Katis (2007) explored the effects of angular position and velocity on functional AR-ratios. The authors found that the effect of angular position on functional AR-ratios was significant. Additionally, they showed that functional AR-ratios were not constant during the ROM. At more extended knee joint positions, the knee flexor torque is progressively increased to prevent excessive anterior tibial translation and assist in the mechanical and proprioceptive roles of the anterior cruciate ligament (Aagaard et al., 2000; Kellis and Katis, 2007). Dehail et al. (2008) also showed a variation in the AR-ratios of the shoulder flexors/extensors and abductors/adductors during the ROM. Moreover, the authors identified a cross point in individuals with paraplegia, defined as the point where agonist and antagonist muscle torques are equal. This cross point is a new parameter that could be developed, but that is not identifiable using the PT method. Recently, based on examination of the rotator muscles of the shoulder, Cozette et al. (2016) showed that the conventional and functional AR-ratios were variable during the ROM with significantly different values depending on the angular ranges. In addition, unlike the AST-ratios, the AR-ratios were significantly different from the classic PT-ratios, especially for the conventional ratio that significantly increased during internal rotation and was significantly lower than the PT-ratio for more than 80% of the ROM. These studies demonstrated that using just the PT-ratios to evaluate muscle balance could lead to misinterpretation. In addition, Cozette et al. (2018) studied the reliability of the AR method for calculating ratios, compared to the PT method. They showed that the AR method (ICC = 0.14–0.85; CoV = 7.1–17.3%) appeared to be more reliable than the PT method (ICC = 0.14–0.75; CoV = 7.0–28.5%) for the conventional and functional ratios at median angular ranges of the shoulder rotation movements.

By limiting analysis to PT values, the potential of isokinetic dynamometers is underexplored. There is a clear lack of research which describes muscular control using sectorial methods. The quantitative sectorial methods seem relevant to assess more precisely the muscle balance or imbalance at each angular sector of joint motion, especially the AST and AR methods. The restoration of muscle function is one of the main targets of rehabilitation programs (i.e., following an injury, immobilization, or surgical intervention). The identification of muscle imbalances associated with specific sectors of the ROM could help optimize functional rehabilitation programs and increase their effectiveness. In addition, the AST or AR ratios could help to better understand the injury mechanisms of athletes and to develop more specific preventive programs for athletic trainers. Further studies are required to assess the reliability of these sectorial approaches and their applications in the exploration of muscle balance. The AST and AR methods could be an excellent alternative to conventional assessments associated with PT and deserve to be further investigated. Although isokinetic assessment does not represent exactly the functional reality, this technology can be optimized. In this perspective, sectorial analysis may enhance current screening methods and help to reflect further the antagonistic muscle capacities during sports and daily activities.

## Author Contributions

MC: writing. P-ML: reviewing. CD: english reviewing. TW: supervisor.

### Conflict of Interest Statement

The authors declare that the research was conducted in the absence of any commercial or financial relationships that could be construed as a potential conflict of interest.
